# Value of endoscopic examination of airways and swallowing in tracheostomy decannulation

**DOI:** 10.1186/s43163-020-00001-9

**Published:** 2020-06-04

**Authors:** Gamal Youssef, Kamal M. Abdulla

**Affiliations:** 1 Phoniatrics Unit, Otolaryngology Department, Faculty of Medicine, Alexandria University, Alexandria, Egypt; 2 Phoniatrics Unit, ENT Department, Dubai Hospital, DHA, Abu Bakr Al Siddiq Street, 7272 Dubai, United Arab Emirates; 3 Otolaryngology, Faculty of Medicine, Al-Azhar University, Cairo, Egypt; 4 Otolaryngology Department, Dubai Hospital, DHA, Dubai, United Arab Emirates

**Keywords:** Tracheostomy, Decannulation, Endoscopic assessment, Tracheostomy decannulation guidelines, FEES, Decannulation failure

## Abstract

**Background:**

Tracheostomy decannulation decision is the major challenge in the clinical management of tracheostomy patients. Little evidence is available to guide the weaning process and optimal timing of tracheostomy tube removal. The purpose of the study was to investigate the value of endoscopic assessment in the tracheostomy decannulation decision.

**Results:**

The study included 154 tracheostomized adult patients. Bedside assessment was done for 112 patients, and the other 42 patients were deceased. The results of bedside assessment lead to successful decannulation in 18 patients (16%), while 94 patients (84%) were unfit for decannulation. The most common cause of unfitness was aspiration and poor swallowing in 41% of patients. The endoscopic assessment was done for 59 patients out of 94 patients that were unfit for decannulation; thirteen patients of them were fit for decannulation (22%). The final status of the patients before discharge was decannulated in 31 cases and 81 patients were discharged with a tracheostomy.

**Conclusions:**

The results indicated the importance of endoscopic assessment in the decannulation decision of tracheostomized patients. A large proportion of patients who are unfit for decannulation by bedside assessment could be fit after endoscopic assessment. Endoscopic assessment is essential particularly in tracheostomized patients who have failed to achieve decannulation through conventional protocols.

## Background

Tracheostomy is one of the most commonly performed surgical procedures in ICU patients by ENT and intensive care unit teams [1]. After weaning from ventilation and improvement of primary indication for tracheostomy, removal of the tracheostomy tube is essential to shorten the hospital stay period and to minimize the burden of tracheostomy care on the patient and his family [2]. Tracheostomy decannulation decision is the major challenge in the clinical management of tracheostomy patients. Little evidence is available to guide the weaning process and optimal timing of tracheostomy tube removal. No validated and standard guidelines are followed by the clinicians in the decannulation decision, and this process is left to expert opinion and institutional guidelines [3–7]. Decannulation decision is crucial because decannulation delay can delay rehabilitation, reduces patient comfort, and is associated with longer hospitalization, higher costs, and more tracheostomy complication. On the other hand, too early decannulation has its risks on the patient and has to be avoided [8, 9].

Many tracheostomy decannulation protocols depend on subjective criteria for decannulation including the strength of cough reflex, ability to expectorate, adequacy of swallowing, condition of the larynx and chest, and patient orientation [10–12].

Decannulation of patients with a prolonged tracheostomy is not as straightforward as tube removal following temporary tracheostomy for acute upper airway obstruction. Patients with multiple medical comorbidities and marginal respiratory status have more risk of decannulation failure [9].

Endoscopic visualization of integrity and function of the airway has been advised to be an objective protocol before decannulation and surgical or medical interventions are often necessary for identified airway obstruction prior to considering decannulation [13, 14].

Fiberoptic endoscopic evaluation of swallowing (FEES) has been proven to be a standardized dysphagia assessment tool, but its use in tracheostomy decannulation protocol with additional endoscopic subglottic and tracheal airway assessment is not firmly established in clinical practice and its use depend on clinical experience. Few studies were done to explore the role of endoscopic assessment in tracheostomy decannulation decision accuracy [5, 9, 15, 16].

We hypothesize that adding endoscopic assessment in tracheostomy decannulation will improve decannulation decision than if it relies only on bedside assessment.

### The aims and objectives of the study

The objective of this retrospective, descriptive study was to compare tracheostomy decannulation decisions based on bedside assessment alone and the decision taken after the endoscopic assessment of upper airway and swallowing and to highlight the value of endoscopic assessment in tracheostomy decannulation success rate.

## Methods

In this retrospective observational study, 154 tracheostomized adult patients from ICU or medical wards in Rashid hospital were included within a 4-year period from 2015 till 2018.

Inclusion criteria: Adult patients with surgical tracheostomy and weaned from the ventilator.

Exclusion criteria: Pediatric population or percutaneous tracheostomy or ventilator-dependent patients.

The following data were collected from the patients’ medical record: age, gender, diagnosis, reasons for tracheostomy, date of tracheostomy, duration of hospital stay, and discharge status either with or without tracheostomy.

Additional data collected included details of bedside assessment used for decannulation assessment and reasons for failed tracheostomy decannulation after bedside assessment. The criteria used in bedside assessment were as follows: patient orientation, cough reflex, frequency of suction through the mouth or through the tracheostomy tube, the swallowing abilities, chest auscultation, tracheostomy tube downsizing or capping, and trial of decannulation and observation.

Patients who referred for ENT and swallowing clinic consultation were subjected to endoscopic assessment protocol, which was done in 3 steps:

1. Standard FEES (fiberoptic endoscopic examination of swallowing), done without local anesthesia spray to avoid impairment of swallowing sensation. The following points were observed:

- Frequency of spontaneous swallowing/minute
- Assessment of laryngeal sensation by touching the epiglottis with the endoscope tip.
- Assessment of salivary pooling in the pyriform fossa and in the supraglottis.
- Assessment of vocal folds for mobility or any organic glottic or supraglottic lesion.
- Standard swallowing assessment of penetration-aspiration scale and residue.

2. Assessment of airway potency after xylocaine spray deep in the throat and introducing the endoscope below the vocal folds to assess sub-glottis till the tracheostomy tube to rule out subglottic or supra-tubal airway obstruction or granuloma.
3. Assessment of lower airway through the tracheostomy tube to visualize the trachea till the carina for signs of infection, tracheostomy tip granulation, and tracheal wall collapse.

If all measures were within the normal range, the patient is considered fit for decannulation, and the tracheostomy tube was removed. The results of the endoscopic assessment were categorized according to fitness for decannulation decision either fit or unfit for decannulation. The causes of unfitness for decannulation after endoscopic assessment were collected.

Statistical analyses were performed with SPSS 20.0 software (SPSS Inc., Chicago, IL). Qualitative variables were measured by descriptive statistics using frequency and percentage. Numerical variables were described using mean and standard deviation. The association between continuous variables was calculated using Student’s *t* test, categorical variables significance differences were measured using the chi-squared test, and two-tailed *P* value < 0.05 was considered significant.

## Results

One hundred fifty-four tracheostomized adult patients (mean age 53.9 ± 23.5 years, 98 males/56 females) from ICU or medical wards were included within a 4-year period. The mean duration of hospital stay was 55 ± 71 days with a range from 9 to 523 days.

The reasons for tracheostomy were prolonged incubation in 71 cases (46%), upper airway obstruction in 34 cases (22.5%), aspiration in 26 cases (17%), prophylaxis in 18 cases (12%), and desaturation in 5 cases (3%) (Table 1).

**Table 1:** Indication of tracheostomy in studied patients

Indication	Frequency	Percentage
Prolonged intubation	71	46
Upper airway obstruction	34	22
Aspiration	26	17
Prophylactic	18	12
Desaturation	5	3

Bedside assessment was done for 112 patients, and the other 42 patients were deceased. The results of bedside assessment lead to successful decannulation in 18 patients (16%), while 94 patients (84%) were unfit for decannulation. The causes of unfitness for decannulation after bedside assessment were desaturation in 24 patients (26%), aspiration and poor swallowing in 39 patients (41%), depressed cough reflex in 17 patients (18%), and lack of orientation in 14 patients (15%) (Table 2).

**Table 2:** Reason of failed decannulation after bedside assessment

Reason	Frequency, number (94)	Percentage
Aspiration and poor swallowing	39	41
Depressed cough reflex	17	18
Desaturation	24	26
Disoriented	14	15

The endoscopic assessment was done for 59 patients out of 94 patients that were unfit for decannulation by bedside assessment (63%). The endoscopic assessment was not done for 35 patients (37%). The results of endoscopic assessment revealed 13 patients were fit for decannulation (22%), 24 patients were unfit due to upper airway obstruction (41%), and 22 cases were unfit due to aspiration (37%) (Table 3).

**Table 3:** Results of endoscopic assessment

Result	Cause	Frequency	Percentage
Unfit for decannulation	Upper airway obstruction	24	41
	Aspiration	22	37
Decannulated	Fit	13	22

**Fig. 1:**
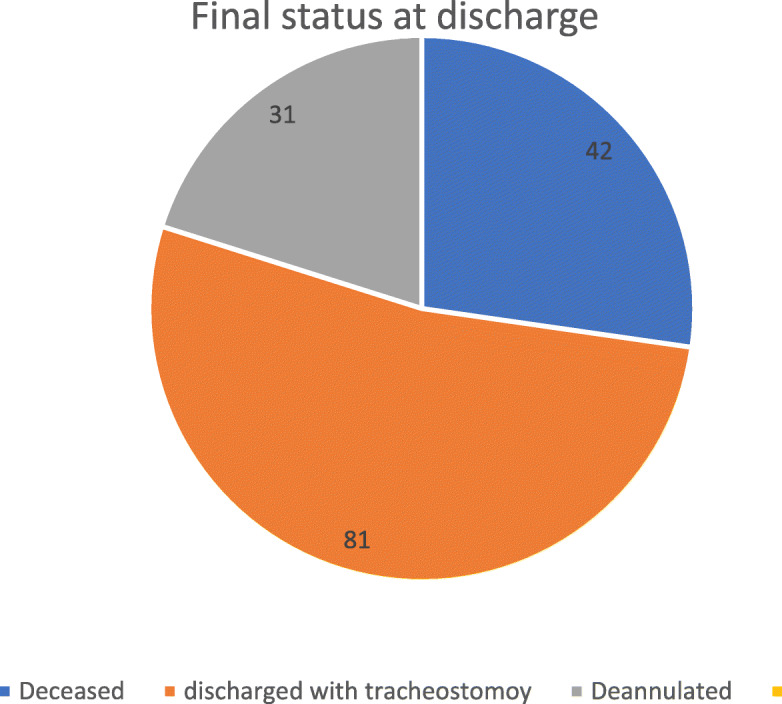
The final status at discharge for tracheostomy patients

The final status of the patients before discharge was decannulated in 31 cases (20.3%) (18 cases after bedside assessment and 13 cases after endoscopic assessment), and 81 patients were discharged with tracheostomy (52.9%) (Fig. 1).

The primary indications of tracheostomy in patients discharged with the tracheostomy tube were prolonged intubation in 41 patients, aspiration in 15 cases, prophylactic in five cases, and desaturation in two cases. The most common reason for tracheostomy in decannulated patients was prophylaxis in 12 cases.

Death during hospital stay happened in 42 cases (26.8%). The primary indications of tracheostomy in ceased patients were prolonged intubation in 21 cases, aspiration in 10 cases, upper airway obstruction in eight cases, desaturation in two cases, and prophylactic in one case.

Table 4 shows the total number of patients with successful decannulation after bedside assessment, and after adding the endoscopic assessment, there was a statistically significant difference in favor of the endoscopic assessment (*P* = 0.000). The mean age of decannulated patients was 39 ± 15 years, and the mean age of the patient discharged with a tracheostomy was 54 ± 24 years; the difference was statistically significant with *P* value 0.007. Regarding the length of hospital stay in both groups, the hospital stay was lower by 12 days in the decannulated patients (58 days) than patients discharged with tracheostomy (46 days), but this difference was not statistically significant (*P* = 0.561).

**Table 4:** Decannulation fitness after bedside assessment and after endoscopy and at discharge

	Bedside assessment (112 pts), no. (%)	Endoscopic assessment (59 pts), no. (%)	Final status after adding endoscopic assessment (122 pts), no. (%)	Chi-square exact sig. (2-sided)
Unfit for decannulation	94 (84)	46 (78)	81 (72)	0.000
Fit for decannulation	18 (16)	13 (22)	31 (28)	0.000

## Discussion

With increasing numbers of tracheostomy patients, more patients will require decannulation [17]. Therefore, it is essential to identify criteria for decannulation fitness to minimize the risk of respiratory compromise and meanwhile improve the clinicians’ confidence in decannulation decisions. Prolonged tracheostomy is associated with morbidity, mortality, and length of stay [6, 18]. Tracheostomy patients are referred for otolaryngology and swallowing consultation to diagnose and manage patients who have failed to achieve decannulation through conventional protocols.

The results of the present study could prove that the endoscopic assessment on tracheostomized patients could improve the tracheostomy decannulation decisions and the rate of success of decannulation.

The total number of decannulated patients after bedside assessment increased by 40% after adding the endoscopic assessment. The present study found a 72% increase in the number of decannulated patients if endoscopy is added than relying on bedside assessment alone. Endoscopy is an objective tool allowing direct visualization of the upper airway for any cause of obstruction and assessment of aspiration. Therefore, the ENT physician is more confident to take decannulation decision than bedside assessment, trial removal, and downsizing.

The total number of unfit patients after doing bedside assessment is 94 patients, the endoscopic assessment was done only for 59 patients, and 13 patients of them (22%) were fit for decannulation. The endoscopic assessment was not done for 35 patients, so if we assumed endoscopy was done, 22% more patients (eight patients) could be fit for decannulation and could be discharged without tracheostomy. So, endoscopic assessment should be part of routine assessment of every patient with tracheostomy before discharge.

Our finding agrees with Warnecke et al. who used endoscopic decannulation protocol in 100 neurologic patients and found 82.8% more patients were successfully decannulated than by relying on bedside assessment alone [9]. Intermediate steps to decannulation were not taken [19]. He speculated that the neuropsychological deficits in neurologic patients can delude physicians into decannulation postponement. Just endoscopic examination allows for the visualization of the laryngeal airway and swallowing functions directly and objectively, irrespective of patient compliance or level of consciousness. They speculated that these functions had really regained better than had been predicted from the initial assessment and earlier and safe decannulation can be done when FEES is applied [9]. Decannulation could be probable in certain cases of patients in a vegetative or in a minimally conscious state after confirming a patent airway, effective cough, and spontaneous swallowing that could be proved by endoscopic assessment and not by bedside assessment [20].

Oakley et al. [21] used Comprehensive Dynamic Airway Assessment (CDAA) in tracheostomy patients’ assessment, which allows a complete upper airway endoscopy, including the subglottis with decannulation under direct vision. He finds significant superiority of the endoscopic approach than using the standard approach in the number of patients decannulated. They concluded that CDAA is an essential diagnostic approach that can improve decannulation outcomes for complex patients with tracheostomies. It requires minimal resources and is a part of the expert nasendoscopic examination skills of the otolaryngologist. CDAA should form an integral part of all decannulation protocols [21].

The most common reason for tracheostomy in decannulated patients was prophylactic, and laryngeal dysfunction is unexpected. Thus, immediate decannulation can be safely done if the airway is proven to be patent by endoscopic assessment.

The study examines a relatively large number of patients of different etiologies in a long duration. The results of the present study indicate the importance of endoscopic assessment for proper management and care of tracheostomized patients. The endoscopic assessment demonstrated to increase the number of patients decannulated and decrease the number of patients discharged with a tracheostomy. The shortages of this study are that it is retrospective with little documented data about the criteria used in bedside assessment and restricted only for surgical tracheostomy.

The age of the decannulated group was significantly lower than the age of the patient discharged with a tracheostomy, and this finding is similar to previous studies as a younger patient has fewer comorbidities and a large percentage of them was prophylaxis tracheostomy [22, 23]. The study found a reduction of hospitalization duration in the decannulated group; even though it was not statistically significant, it is cost-wise important inferences, especially for the growing number of patients with tracheostomy [22].

This study found a large number of patients discharged with the tracheostomy tubes. O’Connor stated that tracheostomy decannulation is an important rehabilitation goal, but cannot always be performed [19]. The severity of the comorbidities and neurological state has a significant influence on decannulation failure [24].

Hales et al. [15] studied the value of adding FEES in tracheostomy weaning and found contradictory results that over a third of all tracheostomized patients that “pass” the bedside swallowing assessment are at risk of aspiration or failed decannulation. This finding supports the use of FEES, but then again, they question that “might FEES prove so sensitive that levels of penetration and aspiration detected may be clinically insignificant, with an unnecessary postponement of weaning and progression to oral intake?” [15]. The results of the present study could answer this question as a large percentage of patients who were unfit for decannulation by bedside assessment were found to be fit for decannulation after endoscopic assessment. Most decannulation protocols require normal swallowing for decannulation fitness, so any abnormality in clinical bedside swallowing assessment can unnecessarily postpone decannulation. Endoscopic airway and swallowing assessment for tracheostomy decannulation examine the patient’s ability to manage his own saliva and spontaneous swallowing, in addition to the successful management of food consistencies. Decannulation could not be done in patients who would really have been keen for decannulation only because they failed in the swallowing study. In the meantime, the patient may not be ready for oral feeding but fit for decannulation. Only an endoscopic assessment can differentiate fitness for oral feeding from fitness for decannulation [9]. Moreover, several studies documented improved dysphagia after decannulation [24, 25].

## Conclusions

The results of the present study indicated the importance of endoscopic assessment in decannulation decision of tracheostomized patients. The use of endoscopic assessment demonstrated to increase the number of patients decannulated and decrease the number of patients discharged with a tracheostomy. Decannulation protocols that depend on bedside assessment alone can preclude decannulation in a large number of patients; therefore, only an endoscopic assessment can differentiate readiness for oral feeding from fitness for decannulation. The ultimate and most important conclusion from this study is that endoscopic assessment is essential especially in tracheostomized patients who have failed to achieve decannulation through conventional protocols.

## Data Availability

The datasets during and/or analyzed during the current study are available from the corresponding author on reasonable request.

## Abbreviations

FEES: Fiberoptic endoscopic evaluation of swallowing
CDAA: Comprehensive Dynamic Airway Assessment
